# The Effects of Different Dietary and Glycogen-Loaded Two-a-Day Training Approaches on Muscle Damage and Inflammatory Markers for Enhancing Metabolic Efficiency

**DOI:** 10.3390/metabo16090674

**Published:** 2026-09-13

**Authors:** Serdar Şerare, Serkan Paçacı, Necip Arman, Ahmet Karadağ, Anıl Şahin

**Affiliations:** 1 Sivas E Type Closed and Open Penal Institution, General Directorate of Prisons and Detention Houses, Ministry of Justice, Ankara 06659, Türkiye; 2 Erzincan Mustafa Dogan Anatolian High School, Ministry of National Education, Ankara 06640, Türkiye; sswipp@gmail.com; 3 Van Nene Hatun Girls’ Vocational and Technical Anatolian High School, Ministry of National Education, Ankara 06640, Türkiye; neciparman@gmail.com; 4 Department of Physical Medicine and Rehabilitation, Faculty of Medicine, Sivas Cumhuriyet University, Sivas 58140, Türkiye; akaradag@cumhuriyet.edu.tr; 5 Department of Cardiology, Faculty of Medicine, Sivas Cumhuriyet University, Sivas 58140, Türkiye; anilsahin@cumhuriyet.edu.tr

**Keywords:** football players, glycogen, Interleukin-6, creatine kinase, lactate dehydrogenase

## Abstract

**Background/Objective:** Insufficient research has examined the muscle damage and inflammatory effects of training performed in a fasted state with reduced (low) glycogen reserves. **Methods:** This study aimed to investigate the acute effects of exercise sessions performed under fasting (FST) and liquid nutrient-supplemented postprandial (PPD) conditions, as well as with full and reduced body glycogen stores, on muscle damage markers creatine kinase (CK) and lactate dehydrogenase (LDH), and the inflammatory marker interleukin-6 (IL-6). Eleven male amateur football players (mean age 19.91 ± 1.64 years) were included in the study. Participants performed a single 60 min aerobic endurance exercise session at 70% VO_2_max under the liquid nutrient-supplemented PPD condition, and two 60 min sessions with a 60 min recovery interval under 10–12 h FST conditions. Data were analyzed using paired samples *t*-test, ANOVA, Wilcoxon, and Friedman tests, depending on parametric and nonparametric assumptions. **Results:** In pre-exercise (PRE-FE) measurements, no significant differences were observed between the FST and PPD conditions for GLU, CK, LDH, and IL-6 levels. In postexercise (POST-FE) measurements, GLU levels were significantly higher in the PPD condition than in the FST condition (*p* < 0.05), whereas LA, CK, LDH, and IL-6 levels did not differ significantly between the two conditions. In the FST condition, following the second exercise session performed with default reduced glycogen availability (POST-SE), CK levels significantly increased from 279.36 ± 16.7 U/L to 335.63 ± 20.3 U/L, LDH levels from 178.72 ± 38.1 U/L to 208.09 ± 50.7 U/L, and IL-6 levels from 2.60 ± 2.4 pg/mL to 8.69 ± 5.8 pg/mL (*p* < 0.05). In the PPD condition, no significant changes were observed in any of the parameters following a single exercise session (*p* > 0.05). **Conclusions:** The two-a-day training approach performed with default reduced glycogen availability may increase muscle damage and inflammatory responses. These findings are considered as an inference that low-glycogen training should not be implemented during the pre-competition period because of the risk of inducing muscle damage.

## 1. Introduction

Following the findings of Bergström et al. [1], which demonstrated that both diet and exercise intensity can significantly modify glycogen content in skeletal muscles and, consequently, influence exercise capacity, numerous exercise scientists and coaches have started to adopt diverse training periodization strategies and off-field techniques to enhance athletes’ performance and endurance. One such strategy involves training in a fasted state after an overnight fast [2]. Current evidence also suggests that training with reduced liver glycogen reserves after an overnight fast may influence metabolic efficiency [3,4]. Another strategy, although not yet formally named, is the two-a-day training approach with short recovery intervals. This approach aims to deplete muscle glycogen stores through the first training session and, after a one-hour interval before complete recovery, perform the second session with reduced (low) glycogen reserves. Hansen et al. [5] reported that an eight-week two-a-day training protocol produced pathological findings indicative of muscular adaptations. It is also thought that commencing training with reduced glycogen levels may further activate lipolysis [6,7,8] and potentially lead to a state of reduced glycogen dependency. Although Hansen et al. [5] implemented two-a-day training in the form of resistance exercise, the literature demonstrates that this approach has also been applied as aerobic endurance training—either at varying intensities or at a fixed percentage of VO_2_max—with recovery intervals ranging from 60 to 105 min [5,9,10,11,12,13,14].

Although it has been investigated whether strenuous two-a-day training approaches performed in a fasted state or with default reduced glycogen availability further activate lipolysis and reduce glycogen dependence, there is a lack of research regarding whether these approaches induce muscle damage or have an inflammatory effect. The literature reports that exercise-induced muscle damage occurs in response to intense exercise, particularly after unfamiliar or novel exercise types. Exercise-induced muscle damage is characterized by disruption of myofibrillar integrity, decreased muscle strength, muscle soreness, and restricted range of motion. Increased cellular permeability resulting from cell membrane damage leads to the leakage of enzymes such as creatine kinase (CK) and lactate dehydrogenase (LDH), which are biochemical markers of muscle damage, as well as muscle proteins such as myoglobin (Mb), into the bloodstream. All these inflammatory processes may lead to decreased performance and consequently negatively affect subsequent training performance [15]. Intense exercise also causes elevations in a range of pro- and anti-inflammatory cytokines, their inhibitors, and chemokines. Interleukin-6 (IL-6) is a cytokine that is an important indicator of inflammatory processes [16]. Whether different exercise modalities can reduce inflammatory marker levels is still debated. While IL-6 serves as a crucial marker for inflammation, it also plays a role in promoting the release of glucose from the liver and in the breakdown of fats during physical activity. In marathon runners, the level of IL-6, which is generated within the skeletal muscle, can surge by as much as 100 times after a race [17]. Symptoms of exercise-induced muscle damage typically manifest within the first 24 h. The assessment period ranges from immediately post-exercise to within the first seven days, with biochemical markers being the most commonly used indicators [15]. Although studies have examined the effects of exercise performed at varying intensities on biochemical parameters such as CK, LDH, and IL-6, which are indicators of muscle damage, there is a paucity of research investigating whether aerobic endurance exercise performed with reduced (low) glycogen reserves in the fasted state (FST), where liver glycogen is low) induces muscle damage within a short timeframe that could negatively affect subsequent training performance. Football players, who use both aerobic and anaerobic energy systems interchangeably [18,19], perform aerobic endurance exercise for 90 min, consisting of two 45 min halves, and sometimes exceeding 120 min with extra time. Additionally, Muslim football players train and compete while fasting during the month of Ramadan. Therefore, football players are considered an appropriate population for examining such physiological variations. This study aimed to examine the acute effects of aerobic endurance training on biochemical markers of muscle damage when liver glycogen reserves are low (FST), in postprandial conditions with liquid nutrient supplementation (PPD), and with partially depleted body glycogen stores. This study contributes to our understanding of how pre-exercise energy availability affects the magnitude of muscle damage. In particular, it may demonstrate the short-term effects of training performed in a fasted state versus following liquid nutrient intake on muscle tissue, thereby facilitating the development of more appropriate training and nutritional strategies for athletes in the future.

Given the limited number of studies in the literature examining how these effects differ across varying glycogen levels, this study on amateur male football players hypothesizes that aerobic endurance exercise may exert differential effects on biochemical markers of muscle damage, depending on body glycogen status.

## 2. Materials and Methods

### 2.1. Study Design

Our experimental research design was modeled on the double-training session protocols of Şerare et al. [9], which were conducted at a fixed intensity of 70% of VO_2_max under fasted and fed conditions, as well as with full and default reduced glycogen availability levels. Some of the authors of the present study were also among the co-authors of that earlier work. Our study was conducted at the Cardiopulmonary and Rehabilitation Unit of Cumhuriyet University. The participants visited our unit three times at one-week intervals. All tests and measurements were performed at the same time of day (±1 h), between 09:00 and 13:00. To ensure that the two training protocols were independent and did not influence each other, the FST and PPD trials were conducted one week apart and in a fixed order (FST first, followed by PPD). Nevertheless, the participants were randomly called for their testing sessions, and only two or three participants were tested per day. This approach was intended to minimize any potential learning, adaptation, or order effects between participants.

**Figure 1:**
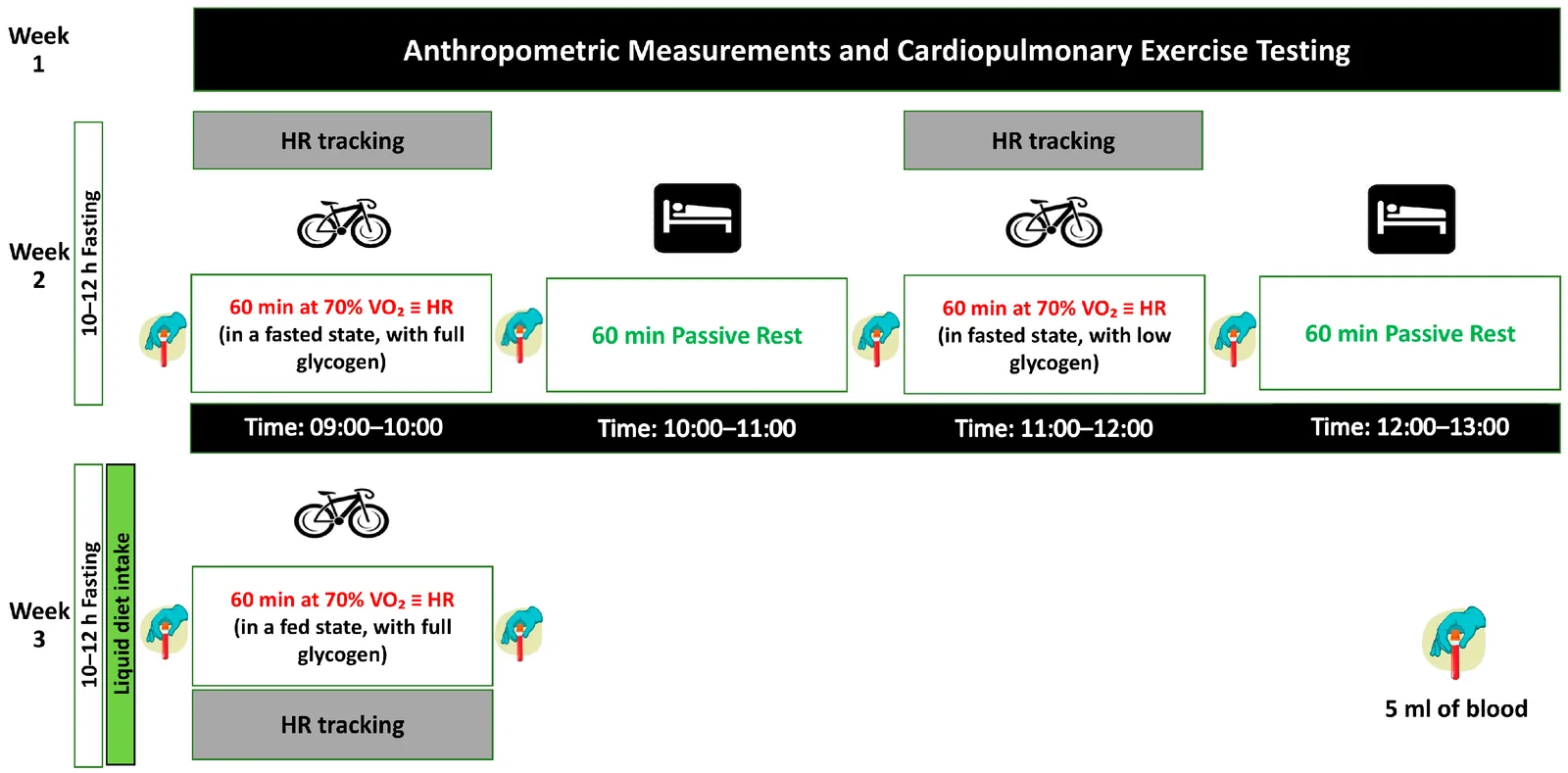
Study Design. HR: Heart Rate, VO_2_max (ml kg^−1^ min^−1^): Maximum Amount of Oxygen (milliliters) Used Per Minute by 1 kg of muscle, VO_2_max 70% ≡ HR: Heart Rate Corresponding to 70% of Maximum Oxygen Utilization, HR ≡ power (watts) Corresponding to Heart Rate, ml: milliliters.

In the first week, assessments were conducted to evaluate anthropometric data, body composition, and aerobic capacity. In the second week, under fasting (FST) conditions established by a 10–12 h overnight fast (placebo) [20], participants performed the first 60 min aerobic endurance exercise session (FE) while liver glycogen stores were partially depleted, but body glycogen stores remained full. Following a 60 min passive recovery period in the supine position, they performed the second 60 min aerobic endurance exercise session (SE) under conditions where both liver glycogen stores (due to the 10–12 h fast) and body glycogen stores were partially reduced (low). In the third week, to enable comparison of biochemical variables between aerobic endurance exercise performed under FST and PPD conditions, participants performed a single 60 min aerobic endurance exercise session under liquid nutrient-supplemented (PPD) conditions with full body glycogen stores with the liquid meal consumed two hours prior to exercise. (Figure 1). It is well established that a 10–12 h fast reduces liver glycogen stores [21], and the partial reduction (depletion) of body glycogen stores through the first exercise session has been confirmed in recent studies conducted with elite male cyclists [13]. Blood samples were collected from all participants before and after each exercise session. The acute effects of aerobic endurance training performed under FST and PPD conditions, as well as with full and partially reduced body glycogen reserves, on biochemical markers indicative of muscle damage and inflammatory response were evaluated through statistical analysis of numerical data obtained from the blood samples. The study protocol is shown in Figure 1.

### 2.2. Participants

The study group initially comprised 12 healthy adult male amateur football players actively competing in local amateur leagues. However, one participant was unable to complete the second experimental exercise session and was subsequently withdrawn from the study. Accordingly, the study was completed with 11 participants with a mean age of 19.91 ± 1.64 years (Table 1). Football players who actively used bicycles in their daily lives were prioritized. For the inclusion of trained amateur football players, the primary selection criteria were the establishment of a homogeneous group in terms of VO_2_max levels and sufficient fitness capacity to complete a 60 min ergometer training session. As aerobic capacity, anaerobic power, and lower extremity strength are essential for football players regardless of playing position, no position-based differentiation was made [22]. Athletes who did not meet the inclusion criteria were excluded from the study; these included those with less than 5 years of training experience, those not actively engaged in football, those who had not performed football-specific training within the last 8 weeks, those with neurological, hormonal, or respiratory system disorders, and those who had sustained a lower extremity injury within the previous 6 months. All participants provided written informed consent to participate in the study after being thoroughly informed about the study’s objectives, the procedures to be applied, and the potential risks involved. Participants were assured that their data would be kept confidential and that they had the right to withdraw from the study at any stage without providing any justification. This study was conducted in accordance with the procedures set forth in the Declaration of Helsinki and was approved by the Sivas Cumhuriyet University Health Research Ethics Committee (date: 12 June 2025, Approval No 2025-06/14).

### 2.3. Anthropometric and Body Composition Assessment

Participants’ height was measured using a SECA 213 stadiometer (Birmingham, UK) with 0.1 cm precision, while participants were dressed in light clothing, such as shorts and T-shirts. Bioelectrical Impedance Analysis (BIA) was employed to assess body composition using a TANITA BC 601 model (Tokyo, Japan) obtained from the Kozmopark Company. BIA calculates parameters, including body weight (kg), fat mass (%), muscle mass (kg), and body mass index (kg/m^2^), by utilizing pre-entered personal data and assessing the body’s electrical conductivity [23] (Table 1).

### 2.4. Cardiopulmonary Exercise Testing (CPET) and Determination of Exercise Intensity

To determine aerobic capacity, the Astrand bicycle ergometer test protocol was administered. The experiment utilized a Monark LC 6 cycle ergometer (Monark Exercise AB, Vansbro, Sweden) [24]. Oxygen (O_2_) consumption was tracked with a Cosmed Quark CPET device (Rome, Italy) throughout the procedure. Heart rate monitoring was conducted using a REF: D41480 ANT+ chest strap integrated with the Cosmed Quark CPET system. Data were collected using a Cosmed Quark CPET gas analyzer [25]. Expired air measurements were performed using a metabolic system (COSMED Quark CPET, Italy) obtained from the ELSA Company. Peak VO_2_ was identified as the maximum oxygen consumption reached in the last 30 s of the test. According to the Astrand protocol, after a 5 min warm-up at 50 Watts and 60 rpm, the workload was set to 100 Watts, with an increase of 50 Watts every two minutes. The participants continued until they reached exhaustion. The participants were verbally encouraged to exert maximal effort [26]. The test was terminated by the operator when VO_2_ or heart rate failed to increase with increasing speed/workload, when respiratory rate exceeded 45 breaths per minute, or when participants indicated that they could not continue [27]. Upon completing the Astrand protocol, the CPET data were used to identify the workload that equated to 70% of VO_2_max, which was then established as the intensity level for the exercise sessions [28,29,30] (Table 1).

### 2.5. Pre-Exercise and During-Exercise Dietary Management

To ensure glycogen and energy balance, participants were asked to refrain from intense training for 48 h prior to the test days, minimize caffeine consumption and avoid alcoholic beverages during the last 24 h, and pay attention to their sleep patterns (at least 8 h of sleep) the night before each exercise day [31]. Additionally, they were instructed to avoid making significant changes to their diet throughout the study period and consume the same types of foods on the day preceding each exercise session [13]. The participants were provided with calculated diets. Written instructions were provided regarding the timing of intake and the prohibition of consuming anything else. The last evening meals before the FST and PPD exercise days were standardized to provide an average of 995.39 ± 68.63 kcal energy. Care was taken to ensure that the meal consisted of 55–65% carbohydrates, 30% fat, and 12–15% protein [32]. The caloric values of the last evening meals were determined based on the basal metabolic rate (BMR), daily energy requirements, and 25% of the calculated daily caloric needs. For male football players aged 18–30 years, BMR was calculated using the following formula: BMR = (15.3 × Body Weight) + 679 = kcal. Daily energy requirements were calculated using the following formula: BMR × activity factor = BMR × (1.6 or 2.4) = kcal/day [33] (Table 1). In the postprandial (PPD) condition, participants consumed a liquid meal two hours before exercise, calculated as kg/10 kcal based on body weight, containing 55% carbohydrate, 30% fat, and 15% protein (Nestlé Türkiye Gıda A.Ş., Istanbul, Turkey) [9]. On the fasting (FST) exercise day, participants consumed a placebo beverage (sucralose and water) matched to be indistinguishable in volume and taste [20] and performed the experimental exercise after a 10–12 h fast. During the one-hour rest interval between the two exercise sessions, participants were allowed to consume water up to a maximum of 500 mL [13] (Table 1).

### 2.6. Aerobic Endurance Exercise

For aerobic endurance exercise, a Monark 928 E bicycle ergometer (Monark Exercise AB, Sweden) was used. The exercise intensity was set at a workload corresponding to 70% of VO_2_max, with the pedaling rate was maintained at 70 rpm [28,29,30]. Heart rate (HR) was continuously monitored using a Polar H1 heart rate sensor chest strap (Polar Electro Oy, Kempele, Finland) integrated with a Polar FT80 wristwatch [34]. Following the first week, during which anthropometric measurements, body composition, and aerobic capacity were determined, participants arrived at the laboratory after a 10–12 h fast in the second week and performed a 60 min aerobic endurance exercise session under the (FST) condition. Following a brief recovery period in the supine position, the same exercise protocol was repeated for an additional 60 min. The purpose of this protocol was to reduce muscle glycogen stores during the initial 60 min exercise bout and subsequently perform a second exercise bout with partially depleted glycogen stores [35]. During the third week of the experimental protocol, participants consumed the liquid nutritional preparation under the postprandial drink (PPD) condition and performed a single 60 min aerobic endurance exercise session.

### 2.7. Biochemical Sample Collection and Measurement

Trained staff collected blood samples from the antecubital vein of each participant at four different time points, both before and after the initial exercise sessions. For lactate (LA) analysis, 5 mL of arterialized blood gas samples were collected in heparinized syringes, whereas venous blood samples for the analysis of metabolite glucose (GLU), cytokine interleukin-6 (IL-6), and enzymes creatine kinase (CK) and lactate dehydrogenase (LDH) were collected in 5 mL syringes and subsequently transferred into 5 mL gel separator tubes for serum analysis. Venous blood samples were transported to the biochemistry laboratory and centrifuged at 3500 rpm and 4 °C to separate the plasma and serum fractions. Blood gas samples were analyzed using a blood gas analyzer (ABL800™, Radiometer, Copenhagen, Denmark). The concentrations of glucose, IL-6, CK, and LDH were measured using a fully automated clinical biochemistry analyzer (Cobas 6000; Roche Hitachi, Mannheim, Germany) [36].

### 2.8. Statistical Analysis

The required sample size was calculated using G*Power 3.1.7 software (Universität Kiel, Germany) [37]. Based on the baseline and post-exercise CK values reported for the leg press condition by Chycki et al. [38]. (170 ± 80 U/L and 262 ± 118 U/L, respectively), an effect size of Cohen’s d = 0.882 was calculated. Using the criteria of α = 0.05, β = 0.20, and a statistical power of 1 − β = 0.80, a minimum of 10 participants was determined to be required. The resulting actual power of the test was found to be *p* = 0.823. Moreover, this sample size is consistent with previous studies that measured pre- and post-exercise CK and LDH levels. [38,39,40]. All statistical analyses were performed using SPSS version 23.0 (IBM Corp., Armonk, NY, USA). The normality of the data distribution was assessed using the Shapiro–Wilk test, and the homogeneity of variances was evaluated using Levene’s test. When parametric assumptions were met, comparisons between two measurements were performed using the paired-samples *t*-test, whereas comparisons involving more than two measurements were conducted using repeated-measures analysis of variance (ANOVA), followed by Bonferroni-adjusted post hoc tests. When the assumptions for parametric analyses were not satisfied, the Wilcoxon signed-rank test was used for comparisons between two measurements, and the Friedman test was used for comparisons involving more than two measurements. In every analysis, a *p*-value of less than 0.05 was considered statistically significant.

## 3. Results

### 3.1. Training Performed Under FST and PPD Conditions

When the comparisons between the FST and PPD conditions in Table 2 were examined, in the PRE-FE measurements, LA levels were significantly higher in the PPD condition than in the FST condition (*p* < 0.05). However, the GLU, CK, LDH, and IL-6 levels did not show significant differences between the two conditions (*p* > 0.05). In the POST-FE measurements, GLU levels were significantly higher in the PPD condition than in the FST condition (*p* < 0.05), whereas no significant differences were observed between the two conditions for LA, CK, LDH, and IL-6 levels (*p* > 0.05).

### 3.2. Training with Full and Default Reduced Glycogen Availability

As shown in Table 3, repeated-measures analysis of the exercise trials performed under fasting conditions (FST) revealed significant differences. LA levels significantly increased from pre-FE to post-FE, significantly decreased from post-FE to pre-SE, and significantly increased again from pre-SE to post-SE (*p* < 0.05). However, GLU measurements did not show any significant differences across any of the time points (*p* > 0.05). IL-6 levels were found to increase significantly between the following time points: pre-FE vs. post-FE, pre-FE vs. pre-SE, pre-FE vs. post-SE, post-FE vs. post-SE, and pre-SE vs. post-SE (*p* < 0.05). For CK and LDH measurements, a significant increase was observed only between pre-SE and post-SE (*p* < 0.05).

## 4. Discussion

This study aimed to investigate the acute effects of aerobic endurance training on muscle damage and inflammatory markers under two different conditions: fasting (FST), characterized by low hepatic glycogen reserves, and a liquid nutrient-supplemented state (PPD). Training sessions were performed with either full or partially reduced body glycogen reserves. One of the most notable findings was the significant increase in CK, LDH, and IL-6 levels after the second exercise session performed with default reduced glycogen availability in the FST condition. In contrast, no significant changes were observed after a single exercise session under the PPD condition. These findings suggest that a two-session training approach with default reduced glycogen availability reserves induces greater metabolic stress in terms of muscle damage and inflammatory response.

### 4.1. Lactate (LA) Under FST and PPD Conditions and During Two-Bout Training

When LA levels, which were examined to monitor exercise intensity and metabolic acidosis, were compared between the FST and PPD conditions, pre-FE measurements were found to be significantly higher in the PPD condition (1.40 ± 0.34 mmol/L) than in the FST condition (1.06 ± 0.37 mmol/L) (*p* < 0.05) (Table 2). This difference may be explained by the fact that liquid nutrient intake stimulates glucose uptake by increasing insulin response, thereby accelerating glycolytic flux. However, the absence of a significant difference in LA levels during post-FE measurements suggests that the exercise-induced LA response was of similar magnitude under both conditions and that the body’s LA buffering capacity was preserved. Under the FST condition, LA increased significantly from 1.06 ± 0.37 mmol/L at pre-FE to 3.82 ± 1.72 mmol/L at post-FE (*p* < 0.05), decreased to 1.46 ± 0.99 mmol/L at pre-SE after a 60 min rest interval, and then increased significantly again to 2.81 ± 1.05 mmol/L at post-SE following the second exercise bout (*p* < 0.05) (Table 3). These findings indicate that during aerobic endurance exercise performed at 70% VO_2_max, the anaerobic contribution increases as glycogen stores become depleted, although the anaerobic threshold is not exceeded during this exercise.

### 4.2. Glucose (GLU) Under FST and PPD Conditions and During Two-Bout Training

The primary rationale for monitoring GLU levels in our study was the possibility that alterations in glycogen stores might influence muscle damage and inflammatory response. No significant difference in GLU levels was observed between the FST and PPD conditions at pre-FE, but post-FE measurements revealed that GLU levels in the PPD condition (100.36 ± 8.44 mg/dL) were significantly higher than those in the FST condition (88.27 ± 8.96 mg/dL) (*p* < 0.05) (Table 2). In the FST condition, although non-significant decreases were observed in already low GLU levels (88.36 ± 10.67 mg/dL) after exercise with both full and default reduced glycogen stores, glucose concentrations were generally maintained (Table 3). Van Proeyen et al. [41] similarly reported that exercise performed under fasting conditions did not induce significant changes in blood glucose utilization. This is supported by previous studies that demonstrated that hepatic gluconeogenesis and glycogenolysis remain active during fasting, thereby preserving blood glucose concentrations [9]. Under low glycogen conditions, increased activity of Pyruvate Dehydrogenase Kinase 4 (PDK4) suppresses the Pyruvate Dehydrogenase (PDH) complex, thereby reducing carbohydrate oxidation and enhancing fat oxidation. In addition, increased muscle contraction associated with default reduced glycogen availability stores activates adenosine monophosphate-activated protein kinase (AMPK), which stimulates insulin-independent Glucose Transporter 4 (GLUT4) translocation and sustains glucose uptake [42]. Under fasting conditions, where hepatic glycogen reserves are diminished, ongoing gluconeogenesis contributes to the maintenance of blood glucose levels in glucose-dependent tissues, particularly the brain [43]. John et al. [44] also reported that following an ultramarathon, hepatic glucose production increases through suppressed insulin secretion and elevated glucagon levels, thereby preserving blood glucose concentrations. However, Small and Margolis [42] noted that glucose tolerance and insulin sensitivity may decrease after approximately 48 h of fasting. While this supports the possibility that muscle damage and inflammatory responses may be further exacerbated by prolonged fasting, we consider that the 10–12 h fasting period applied in our study likely remained below the threshold for the onset of adverse metabolic effects. Nash et al. [45] reported that glycogen depletion is associated with both exercise duration and intensity and that intramuscular glycogen content may serve as a key biochemical determinant of IL-6 production.

### 4.3. Interleukin-6 (IL-6) Under FST and PPD Conditions and During Two-Bout Training

IL-6 is a cytokine secreted from the skeletal muscle during exercise, exhibiting both metabolic regulatory and pro-inflammatory properties [46]. The assessment of IL-6 levels is important for elucidating the relationship between metabolic stress induced by low glycogen conditions and muscle damage, as well as the inflammatory response. In our study, when comparing the FST and PPD conditions, no significant differences were observed in IL-6 levels at either pre-FE or post-FE measurements (Table 2). The lack of change in IL-6 levels under the PPD condition may be explained by the fact that sufficient glycogen stores support intramuscular glucose utilization during exercise, thereby reducing the need for IL-6-mediated metabolic regulation. In contrast, under the FST condition, IL-6 levels increased significantly from 2.60 ± 2.4 pg/mL at pre-FE to 4.52 ± 3.2 pg/mL at post-FE (*p* < 0.05), to 4.46 ± 2.1 pg/mL at pre-SE (*p* < 0.05), and reached their highest level at post-SE with 8.69 ± 5.8 pg/mL (*p* < 0.05) (Table 3). The highest IL-6 level observed, particularly at post-SE, indicates that performing the second exercise bout with default reduced glycogen availability imposes additional metabolic stress. Gavin et al. [47], in a study involving 12 active male individuals with a mean age of 23 ± 4 years, reported that plasma IL-6 responses following eccentric exercise were similar under normal and default reduced glycogen availability conditions. This discrepancy may be attributed to differences in exercise protocol, exercise intensity, and participant characteristics. In contrast, Hamilton et al. [48], in a study involving 14 adults with a mean age of 26.8 ± 6.7 years, reported that IL-6 levels increased after 45 min of evening exercise (at 65–70% VO_2_peak) under both fasting (water) and fed (sugared milk) conditions, and that nutritional status did not significantly alter the IL-6 response. While this finding supports the IL-6 increase observed after FST in our study, it also suggests that the additional increase observed after the second exercise bout may be related to the metabolic stress imposed by the default reduced glycogen availability. Several molecular mechanisms underlie the increase in IL-6 under low glycogen conditions. Reduced carbohydrate availability activates adenosine monophosphate-activated protein kinase (AMPK) and p38 mitogen-activated protein kinase (p38 MAPK) signaling pathways, thereby increasing IL-6 gene expression and release [45,49]. Nieman et al. [50], in a study involving 20 male cyclists with a mean age of 38.4 ± 6.0 years, demonstrated that muscle glycogen decreased by 77.2 ± 17.4% following a 75 km time trial performed at 69.6% VO_2_max. However, they found that glycogen reduction alone did not fully explain the IL-6 response and that a moderate correlation existed between muscle glycogen and plasma IL-6 (r = −0.462, *p* = 0.040), with muscle damage and other metabolic stress factors also playing a role in this response. Şerare et al. [9], in a study involving 14 amateur male football players, reported that increased cortisol levels under low glycogen conditions may support glucose homeostasis through gluconeogenesis, while also increasing IL-6 release via metabolic stress. Collectively, these findings suggest that the IL-6 increase observed after the second exercise bout under the FST condition in our study is not solely attributable to glycogen depletion but may represent a response to the metabolic stress induced by default reduced glycogen availability. In our study, the IL-6 increase under the FST condition was accompanied by significant elevations in CK and LDH levels, indicating that double-bout exercise performed with low glycogen increases the risk of muscle damage. Tomazoni et al. [51], in a study involving 22 male football players with a mean age of 18.85 ± 0.61 years, demonstrated that pre-exercise photobiomodulation suppressed muscle damage and inflammatory responses by reducing CK, LDH, and IL-6 levels together. Furthermore, the authors reported that systemic increases in muscle damage markers following a football match may trigger an inflammatory response by increasing cytokine levels, such as TNF-α and IL-6, and that IL-6 is one of the most potent mediators of the acute phase of the inflammatory response in skeletal muscle. IL-6 is also reported to stimulate hepatic glucose production and lipolysis during exercise [52]. Therefore, the elevated IL-6 levels observed post-SE under the FST condition in our study may be considered as part of the metabolic adaptation to default reduced glycogen availability. Moreover, Nash et al. [45] reported that carbohydrate intake suppresses IL-6 production, whereas low muscle glycogen potentiates the IL-6 response. They also noted that the classical IL-6 signaling pathway, which predominates during exercise, is associated with anti-inflammatory effects, whereas the trans-signaling pathway is a key determinant of the pro-inflammatory response. Grau et al. [53], in a study involving 12 individuals (9 male, 3 female) with a mean age of 48.3 ± 6.6 years, reported that IL-6 levels increased significantly following a 230 km ultramarathon (*p* = 0.0002). The researchers reported that glycogen depletion is an important metabolic stimulus, but that muscle damage and stress-mediated mechanisms also enhance IL-6 release. They further reported that IL-6 initiates a systemic inflammatory response by stimulating C-Reactive Protein (CRP) synthesis and contributes to anti-inflammatory processes by increasing IL-10 release. When these findings are evaluated together, it appears that the IL-6 increase observed after the second exercise bout under the FST condition in our study is not solely due to glycogen depletion but rather reflects a multifactorial response potentiated by the metabolic stress induced by default reduced glycogen availability. Chycki et al. [38] reported that IL-6 levels significantly increased from 1.58 ± 1.77 pg/mL to 2.49 ± 1.86 pg/mL (*p* = 0.004) following leg press exercise in 11 male participants with a mean age of 23 ± 2 years. The authors suggested that this increase might be related to glycogen depletion rather than muscle damage. This finding supports the IL-6 increase observed after the second exercise session with reduced glycogen under the FST condition in our study (2.60 ± 2.4–8.69 ± 5.8 pg/mL). In both studies, reduced glycogen availability appears to enhanced IL-6 responses. These observations collectively reveal that metabolic stress, rather than muscle damage alone, may be the primary driver of the IL-6 response under low-glycogen conditions.

### 4.4. Creatine Kinase (CK) and Lactate Dehydrogenase (LDH) Under FST and PPD Conditions and During Two-Bout Training

CK and LDH are among the most commonly used biochemical markers of muscle membrane damage [15]. John et al. [44] state that increases in muscle damage markers are associated with exercise-induced muscle fiber disruption and the leakage of intracellular enzymes into circulation. Soler-López et al. [54] noted that LDH provides complementary but non-specific information regarding muscle membrane disruption and metabolic stress, and that CK should be interpreted in conjunction with symptoms and recent exercise load. In our study, the absence of significant changes in CK and LDH levels following a single exercise session performed under the PPD condition suggests that sufficient glycogen stores may be effective in preserving muscle membrane integrity. The lack of significant differences in CK and LDH levels between the FST and PPD conditions at both pre-FE and post-FE measurements (Table 2) indicates that glycogen status did not influence CK and LDH levels after a single 60 min session of aerobic endurance exercise. In contrast, under the FST condition, CK levels increased significantly between pre-SE (279.36 ± 16.7 U/L) and post-SE (335.63 ± 20.3 U/L) (*p* < 0.05), and LDH levels also increased significantly between pre-SE (178.72 ± 38.1 U/L) and post-SE (208.09 ± 50.7 U/L) (*p* < 0.05) (Table 3). The lack of significant differences in CK and LDH levels between post-FE and post-SE indicates that post-second exercise values did not differ from post-first exercise values. However, the key finding was a significant increase in CK and LDH levels from pre-SE to post-SE (*p* < 0.05). This increase provides evidence that a second exercise bout performed with default reduced glycogen availability reserves significantly exacerbates muscle damage. Although no significant increase was observed at post-FE, the significant increase at post-SE indicates that glycogen depletion is a critical factor in the development of muscle damage. This suggests that although a two-bout training approach may be beneficial for muscle adaptation [5], it has the potential to increase muscle damage in the short term. Soler-López et al. [54] noted that the time course of post-exercise CK elevation is delayed, with values beginning to rise several hours after exercise and peaking between 24 and 72 h. Li et al. [55] state that LDH levels are associated with biochemical adaptations to physical load and muscle status, while CK is related to both exercise intensity and duration. In our study, the increases in CK and LDH observed after the second exercise bout with default reduced glycogen availability under the FST condition also reflected the effects of exercise duration and intensity on muscle damage. Considering that blood samples were collected immediately after exercise, we anticipate that the observed CK increases may become even higher during the recovery period and that the true extent of muscle damage may become more apparent in the subsequent hours. Marqués-Jiménez et al. [56] stated that the eccentric component of football matches can lead to exercise-induced muscle damage, resulting in performance decline and increases in CK, LDH, and IL-6. The researchers indicated that post-match CK elevations can remain elevated for 24, 48, and 72 h, while LDH elevations may increase further after 48 h and persist up to 72 h. Matkovic and Devrnja [57] demonstrated significant increases in CK levels from 291.88 ± 166.87 U/L to 905.86 ± 759.38 U/L (*p* < 0.001) and in LDH levels from 208.67 ± 43.74 U/L to 261.86 ± 60.04 U/L (*p* < 0.001) following a 90 min match in 43 young male football players with a mean age of 16.8 ± 1.06 years. In our study, exercise was performed on a cycle ergometer at a fixed workload corresponding to 70% of the VO_2_max. Although cycling exercise is more concentric and performed at a constant intensity compared to the eccentric loading in football matches, the total exercise duration of 120 min and the performance of the second bout with default reduced glycogen availability similarly triggered muscle damage. We believe that despite cycling exercise imposing lower mechanical stress than a football match, the metabolic stress induced by glycogen depletion plays a crucial role in the development of muscle damage. The significant increases (*p* < 0.05) in CK, LDH, and IL-6 levels observed after the second exercise bout with reduced glycogen under the FST condition in our study are consistent with the literature on exercise-induced muscle damage and inflammatory responses in football players. These findings indicate that low-glycogen two-bout training may increase muscle damage and inflammatory responses, underscoring the importance of adequate recovery time (48–72 h) [56]. Li et al. [55] reported significant increases in CK and LDH (*p* < 0.001) following a 400 km ultramarathon in 16 male ultramarathon runners with a mean age of 40.3 ± 7.0 years (pre-race CK: 195.3 ± 123.0 IU/L, LDH: 203.2 ± 42.1 IU/L; post-race CK: 2518.5 ± 2276.2 IU/L, LDH: 511.8 ± 249 IU/L). Hearris et al. [58] proposed a glycogen threshold concept, suggesting that the beneficial adaptations of low-glycogen training become evident when exercise is commenced with muscle glycogen concentrations below <300 mmol/kg dry weight [58]. Knuiman et al. [49] defined a critical glycogen level (~250–300 mmol/kg dry weight), and falling below this threshold impairs calcium release from the sarcoplasmic reticulum, adversely affecting the contractile mechanism and threatening cell membrane integrity, potentially leading to CK and LDH leakage. In our study, the likelihood that glycogen levels fell below this threshold prior to the second exercise bout under the FST condition explains the significant increases observed in CK and LDH. Duhamel, Perco, and Green [59] demonstrated that cycling exercise at 70% VO_2_peak performed under low glycogen conditions caused more rapid declines in sarcoplasmic reticulum calcium ion (SR Ca^2+^) uptake and release compared to high glycogen conditions (SR Ca^2+^ uptake rate at 30 min: high carbohydrate 2.93 ± 0.10 vs. low carbohydrate 2.23 ± 0.12 µM/g protein/min, *p* < 0.05; at 67 min: high carbohydrate 2.77 ± 0.16 vs. low carbohydrate 2.10 ± 0.12 µM/g protein/min, *p* < 0.05). This finding indicates that aerobic endurance training performed with default reduced glycogen availability may cause mechanical and metabolic stress sufficient to compromise the integrity of muscle cell membranes. Cheng et al. [60] applied a protocol consisting of 60 min of cycling at 60% VO_2_max followed by 6 × 30 s all-out sprints (with 4 min recovery) in 9 recreationally active males with a mean age of 30.0 ± 6.0 years. The researchers noted that glycogen depletion impairs SR Ca^2+^ release, particularly when it occurs from intramyofibrillar pools adjacent to the SR. Córdova Martínez et al. [40] reported that CK and LDH activities increased after each stage in well-trained semi-professional cyclists during successive cycling stages, along with increases in pro-inflammatory cytokines. The researchers stated that repeated successive stages lead to the accumulation of plasma muscle damage markers, lipid peroxidation markers, and pro-inflammatory cytokines, and that this accumulation is likely a consequence of local inflammatory responses in the skeletal muscle. These findings suggest that the significant increases in CK and LDH levels observed after the second exercise bout with default reduced glycogen availability under the FST condition in our study may be the result of the cumulative effect of repeated exercise bouts. John et al. [44], in a 230 km ultramarathon study involving 43 runners (16 female, 27 male), reported significant increases in CK and LDH immediately after the race (*p* < 0.0001). This finding demonstrates that muscle damage markers may exhibit different responses depending on exercise load and duration. Augusto et al. [61] reported that CK levels significantly increased from 460.0 ± 221.0 U/L to 705.5 ± 421.0 U/L and LDH levels from 166.0 ± 33.0 U/L to 240.0 ± 80.0 U/L following a consecutive high-intensity exercise protocol (5 km run, rope climbing, swimming, and similar activity) in male military personnel with a mean age of 23.0 ± 2.0 years, with blood samples collected at 04:00–05:00 a.m. (i.e., under overnight fasting conditions). This study supports our findings by demonstrating that exercise protocols performed under fasting conditions and involving consecutive sessions lead to significant increases in markers of muscle damage. In the same study conducted by Chycki et al. [38] with 11 male participants, it was also reported that CK levels significantly increased from 170 ± 80 U/L to 262 ± 118 U/L (*p* = 0.024) following leg press exercise, and from 159 ± 63 U/L to 284 ± 202 U/L (*p* = 0.014) following bench press exercise. These findings support the increase in CK observed after the second exercise session with reduced glycogen under the FST condition in our study (279.36 ± 16.7–335.63 ± 20.3 U/L). In both studies, high metabolic stress exercise protocols caused significant increases in CK levels. Taken together, these findings indicate that exercise performed under reduced glycogen availability, particularly in consecutive sessions, may acutely compromise the integrity of the muscle membrane.

This study had several limitations. First, as the participants consisted exclusively of amateur male football players, the findings may not be generalizable to professional football players or female athletes. Second, muscle damage markers were assessed solely through blood samples, and invasive methods, such as muscle biopsy or imaging techniques, were not employed. Third, this study only examined acute effects, and prolonged recovery processes were not evaluated. Furthermore, because blood samples were collected immediately post-exercise, our measurement timeline may have significantly underestimated the peak values of delayed markers such as CK and lactate dehydrogenase LDH, which typically peak between 24 and 72 h post-exercise. Consequently, the current findings likely capture only the initial phase of the muscle damage cascade and may not reflect the full magnitude of exercise-induced muscle damage, particularly under low-glycogen conditions. Future studies should incorporate serial blood sampling over an extended recovery period (e.g., 24–72 h) to more comprehensively evaluate the temporal dynamics of these delayed markers. Fourth, no comparisons were made with different exercise modalities or high-intensity interval training (HIIT). Finally, the study was only partially controlled through the use of a placebo beverage (sucralose and water) under FST conditions, as no true placebo control group was included. Another limitation is that the FST and PPD trials were conducted in a fixed order (FST first, followed by PPD) rather than in a randomized or counterbalanced sequence. This prevents us from entirely ruling out a potential period or order effect. However, the one-week washout period between trials may have mitigated this effect to some extent. Nevertheless, the participants were called for their testing sessions in a random order, and only two or three participants were tested per day. Furthermore, a placebo beverage was used under the FST condition to blind the participants to the experimental condition. These approaches were intended to minimize any potential learning, adaptation, or order effects. Nonetheless, these potential limitations should be considered when interpreting our findings.

## 5. Conclusions

This study investigated the acute effects of aerobic endurance training performed under different hepatic glycogen conditions (FST vs. PPD) and with varying whole body glycogen stores (full vs. reduced) on muscle damage and inflammatory markers. Lactate (LA) levels, assessed to monitor exercise intensity and metabolic acidosis, increased significantly during both exercise bouts under the FST condition, whereas no significant differences were observed between the FST and PPD groups at post-FE measurements. Glucose (GLU) levels were significantly higher in the PPD condition than in the FST condition, whereas GLU concentrations were maintained throughout all exercise trials under the FST condition for glucose-dependent tissues. No significant differences were observed in CK, LDH, or IL-6 levels before or after exercise between the PPD and FST conditions. Under the FST condition, while the first exercise bout performed with full glycogen stores did not induce significant changes relative to pre-FE, the second aerobic endurance exercise bout performed with reduced glycogen reserves (post-SE) resulted in significant increases in CK and LDH levels compared to pre-SE. Full glycogen reserves appear to exert a protective effect against muscle damage and inflammatory responses, whereas a two-bout training approach performed under low-glycogen conditions may acutely exacerbate muscle damage. Supporting these findings, IL-6—which is known to play a role in metabolic regulation (gluconeogenesis and lipolysis), was found to increase throughout all exercise bouts under the FST condition, reaching its highest level at post-SE. This finding suggests that training with default reduced glycogen levels may trigger an inflammatory response to metabolic stress. These results indicate that glycogen status should be considered in training periodization and that two-bout training performed with presumably reduced glycogen availability may be strategically used to enhance metabolic efficiency. However, since such a training approach may elevate acute muscle damage markers, it is considered an inference that its implementation during the pre-competition period may pose a risk.

## Figures and Tables

**Table 1:** Anthropometric Characteristics, Training History, Maximal Oxygen Uptake Capacities, and Dietary Caloric Intake Values Before and After the First Exercise Session in the Postprandial State Among Participants (n = 11).

Variables	Mean ± SD	Min	Max
Age (years)	19.91 ± 1.64	18.00	23.00
Training (Years)	8.36 ± 2.42	5.00	12.00
Height (cm)	174.44 ± 5.96	167.00	184.00
Body Weight (kg)	63.71 ± 7.35	53.50	78.20
BMI (kg/m^2^)	21.17 ± 1.22	17.90	23.80
Resting HR (beats/min)	68.63 ± 5.46	58.00	76.00
Max HR (beats/min)	186.09 ± 9.17	165.00	197.00
VO_2_max (ml kg^−1^ min^−1^)	45.12 ± 3.41	40.30	51.70
VO_2_max 70% (ml kg^−1^ min^−1^)	32.75 ± 4.03	28.21	42.70
VO_2_max 70% ≡ HR (beats/min)	153.63 ± 5.62	141.00	159.00
Basal Metabolic Rate (BMR; kcal)	1658.81 ± 113	1497	1875
Daily Calorie Needs (kcal)	3982.27 ± 272	3594	4501
Last Evening Diet (Solid Food, kcal)	995.39 ± 68.63	898.53	1125.28
PPD PRE-FE (Liquid Food, kcal)	648.72 ± 73.72	535.00	782.00
PPD POST-FE (Liquid Food, kcal)	129.70 ± 14.67	107.00	156.00

SD: Standard Deviation, Min: Minimum, Max: Maximum, BMI: Body Mass Index, kg: Kilogram, cm: Centimeter, HR (beats/min): Heart Rate Per Minute, Max HR: Maximum Heart Rate, VO_2_max (mL kg^−1^ min^−1^): Maximum Amount of Oxygen (milliliters) Used Per Minute by 1 Kilogram of Muscle, VO_2_max 70% ≡ HR: Heart Rate Corresponding to 70% of maximum Oxygen Utilization, HR ≡ Power (watts): Power (watts) Corresponding to Heart Rate. PPD: Postprandial, PRE-FE: Before the First Exercise, POST-FE: After the First Exercise.

**Table 2:** Statistical comparison of biochemical parameters obtained from aerobic endurance exercise performed under fasting (FST) and postprandial (PPD) conditions (n = 11).

Pre-First Exercise (PRE-FE)					
Variables	FST (Mean ± SD)	PPD (Mean ± SD)	t	Z	*p*
**LA (mmol/L)**	1.06 ± 0.37	1.40 ± 0.34		−2.490	0.013 *
**GLU (mg/dL)**	88.36 ± 10.67	96.81 ± 14.37	−1.841		0.200
**CK (U/L)**	267.54 ± 14.3	195.45 ± 10.4	1.265		0.235
**LDH (U/L)**	182.81 ± 36.6	176.36 ± 36.42	0.615		0.553
**IL-6 (pg/mL)**	2.60 ± 2.4	2.67 ± 2.8		−0.140	0.889
**Post-First Exercise (POST-FE)**					
**LA (mmol/L)**	3.82 ± 1.72	4.60 ± 1.48		−1.290	0.197
**GLU (mg/dL)**	88.27 ± 8.96	100.36 ± 8.44	−3.818		0.003 *
**CK (U/L)**	291.72 ± 166	211.36 ± 109	1.213		0.253
**LDH (U/L)**	188.09 ± 39.32	200.90 ± 48.95	−0.928		0.375
**IL-6 (pg/mL)**	4.52 ± 3.2	4.85 ± 4.29		−0.153	0.878

Note. Mean ± SD: Mean ± Standard Deviation, LA: Lactic acid, IL-6: Interleukin-6, GLU: Glucose, CK: Creatine kinase, LDH: Lactate dehydrogenase, mmol/L: Millimoles per Litre, pg/mL: Picograms per milliliter, mg/dL: Milligram/Deciliter, U/L: Units per Liter, PRE-FE: Pre-first exercise, POST-FE: Post-first exercise, *p*-value between tests. Significance level: * *p* < 0.05.

**Table 3:** Statistical comparison of biochemical parameters obtained from aerobic endurance exercise performed under fasting conditions (FST) with full and partially reduced body glycogen reserves (n = 11).

	FST	Mean ± SD	*p*	Friedman Test	*p*
**LA** (mmol/L)				PRE-FE < POST-FE	0.0003 *
	PRE-FE	1.06 ± 0.37		PRE-FE PRE-SE	0.261
	POST-FE	3.82 ± 1.72	0.0001 *	PRE-FE < POST-SE	0.0005 *
	PRE-SE	1.46 ± 0.99		POST-FE > PRE-SE	0.0003 *
	POST-SE	2.81 ± 1.05		POST-FE POST-SE	0.075
				PRE-SE < POST-SE	0.016 *
**IL-6** (pg/mL)				PRE-FE < POST-FE	0.0008 *
	PRE-FE	2.60 ± 2.4		PRE-FE < PRE-SE	0.0033 *
	POST-FE	4.52 ± 3.2	0.0001 *	PRE-FE < POST-SE	0.0003 *
	PRE-SE	4.46 ± 2.1		POST-FE PRE-SE	1.000
	POST-SE	8.69 ± 5.8		POST-FE < POST-SE	0.0006 *
				PRE-SE < POST-SE	0.010 *
	**FST**	**Mean ± SD**	***p***	**ANOVA Test**	***p***
**GLU** (mg/dL)					
	PRE-FE	88.36 ± 10.67			
	POST-FE	88.27 ± 8.96	0.282		
	PRE-SE	86.45 ± 8.77			
	POST-SE	84.63 ± 12.33			
**CK** (U/L)				PRE-FE POST-FE	0.580
	PRE-FE	267.54 ± 14.3		PRE-FE PRE-SE	1.000
	POST-FE	291.72 ± 16.6	0.0030 *	PRE-FE POST-SE	0.194
	PRE-SE	279.36 ± 16.7		POST-FE PRE-SE	1.000
	POST-SE	335.63 ± 20.3		POST-FE POST-SE	0.425
				PRE-SE < POST-SE	0.043 *
**LDH** (U/L)				PRE-FE POST-FE	1.000
	PRE-FE	182.81 ± 36.6		PRE-FE PRE-SE	1.000
	POST-FE	188.09 ± 39.32	0.0010 *	PRE-FE POST-SE	0.492
	PRE-SE	178.72 ± 38.1		POST-FE PRE-SE	0.313
	POST-SE	208.09 ± 50.7		POST-FE POST-SE	0.463
				PRE-SE < POST-SE	0.034 *

Note. Mean ± SD: Mean ± Standard Deviation, LA: Lactic acid, IL-6: Interleukin-6, GLU: Glucose, CK: Creatine kinase, LDH: Lactate dehydrogenase, mmol/L: Millimoles per Litre, pg/mL: Picograms per milliliter, mg/dL: Milligram/Deciliter, U/L: Units per Liter, PRE-FE: Pre-first exercise, POST-FE: Post-first exercise, PRE-SE: Pre-second exercise, POST-SE: Post second exercise, *p* value between tests. Significance level: * *p* < 0.05.

## Data Availability

De-identified participant-level data are available upon reasonable request from the corresponding author, subject to the approval of the Sivas Cumhuriyet University Health Research Ethics Committee and applicable data protection requirements.

## Abbreviations

The following abbreviations are used in this manuscript.

AMPK	Adenosine Monophosphate-Activated Protein Kinase
BIA	Bioelectrical Impedance Analysis
BMR	Basal Metabolic Rate
CK	Creatine Kinase
CPET	Cardiopulmonary Exercise Testing
FST	Fasting Condition
GLU	Glucose
GLUT4	Glucose Transporter 4
HIIT	High-Intensity Interval Training
HR	Heart Rate
IL-6	Interleukin-6
LA	Lactate
LDH	Lactate Dehydrogenase
mL	Milliliter
PDH	Pyruvate Dehydrogenase
PDK4	Pyruvate Dehydrogenase Kinase 4
PPD	Liquid Nutrient-Supplemented Postprandial Condition
PRE-FE	Pre-First Exercise
POST-FE	Post-First Exercise
PRE-SE	Pre-Second Exercise
POST-SE	Post-Second Exercise
VO_2_max	Maximal Oxygen Uptake
